# Three-dimensional templating in hip arthroplasty: the basis for template-directed instrumentation?

**DOI:** 10.1007/s00402-020-03394-7

**Published:** 2020-03-10

**Authors:** P. Savov, S. Budde, S. Tsamassiotis, H. Windhagen, M. Klintschar, M. Ettinger

**Affiliations:** 1 Department of Orthopedic Surgery, Hannover Medical School, Anna-von-Borries-Strasse 1-7, 30625 Hanover, Germany; 2 Institute for Forensic Medicine, Hannover Medical School, Carl-Neuberg-Straße 1, 30625 Hanover, Germany

**Keywords:** THA, 3D templating, Anterior femoral offset, Centre of rotation, Template-directed instrumentation, TDI

## Abstract

**Introduction:**

Computed tomography-based three-dimensional models may allow the accurate determination of the center of rotation, lateral and anterior femoral offsets, and the required implant size in total hip arthroplasty. In this cadaver study, the accuracy of anatomical reconstruction was evaluated using a three-dimensional planning tool.

**Materials and methods:**

A total of eight hip arthroplasties were performed on four bilateral specimens. Based on a computed tomography scan, the position and size of the prosthesis were templated with respect to the anatomical conditions.

**Results:**

On average, all parameters were reconstructed to an accuracy of 4.5 mm and lie within the limits recommended in the literature. All prostheses were implanted with the templated size.

**Conclusions:**

The exact anatomy of the patient and the required size and position of the prosthesis were precisely analyzed using a templating software. Based on the present findings, the development of template-directed instrumentation is conceivable using this method. However, further technical features (e.g., navigation or robot-assisted surgery) are required for improved precision for implant positioning.

## Introduction

Careful planning is crucial for the success of total hip arthroplasty (THA), affecting the occurrence of intraoperative and postoperative complications, the recovery process, and the long-term results [7, 25]. Hence, preoperative templating plays a central role. Currently, two-dimensional (2D) templating using conventional X-rays is the gold standard [8, 25, 30]. In the literature, the reported accuracy of preoperative templating in determining the exact size is 35–48% [11, 15, 30]. If the accuracy is extended to predict to one size, the value is increased to 60–94% [15, 25]. Therefore, computed tomography (CT) scans were developed, demonstrating a clearly higher accuracy in size prediction (estimated at 94–100%) [13, 30, 31]. In the present study, we used the “modiCAS|3D^®^–software” (modiCAS GmbH, Erlangen, Germany) for 3D templating based on a preoperative CT scan. Currently, there are no data available in the literature concerning this software. In this study, we evaluated the accuracy of the preoperative 3D templating in determining the size of the implant. Templating of an exact implant may lead to the development of template-directed instrumentation (TDI). Previous studies have performed cost analyses with regard to TDI. The costs for the preparation, sterilization, and packing of the trays may be reduced by more than double [16].

An additional aspect of the preoperative planning is the evaluation of the anatomical femoral offset, the center of rotation (COR) of the hip joint, and the distinction of different types of architectural hip deformities [18]. Restoration of the COR is the main goal of THA, and there are several advantages to this approach. If the anatomical restoration of the COR is achieved, it reduces the risk of dislocation and impingement of bone or soft tissue [12, 19, 26, 28, 34]. Furthermore, the kinematics of the hip, the abductor function, and the patient outcome scores are improved [1, 24, 32, 33]. In addition, there are data describing a decreased wear and long-term loosening [6, 17, 20, 23]. The role of the lateral femoral offset (LFO) in THA is well investigated. Of note, the anatomical reconstruction delivers great benefits [3, 21, 22, 29]. However, evaluation of the LFO using the conventional 2D X-ray is characterized by limitations. For example, the measurement is complicated due to the rotation of the femur neck [22]. The precision of the measurement with a CT scan is markedly superior to that obtained with an X-ray [21, 29]. However, the anterior femoral offset (AFO) is not sufficiently investigated. In addition, from our point of view, no sufficient measuring method of the AFO is currently described. Using a CT-based computer simulation, Hirata et al. demonstrated a positive correlation between the AFO and the range of motion (ROM) of the hip joint [14]. The aim of this study was to evaluate the possibility of TDI, as well as the restoration of the LFO, COR, and AFO through 3D templating.

## Materials and methods

In this experimental study, bilateral THA was performed in four fresh frozen cadavers. The specimens were a complete lower limb from the pelvis (L2/3) to the tips of the toes. They were sourced from Science Care^®^ (Phoenix, Arizona, USA). The only exclusion criterion for the cadavers was a BMI > 28. The quality of the bone was not considered. A preoperative CT scan was performed on the cadavers, following the CT protocol for the templating software “modiCAS|3D^®^”. This protocol includes scans of the pelvis, knee, and ankle. The pelvis is reconstructed using two different fields of view in the axial layers. Initially, a 400-mm bilateral dataset (layer thickness: 2 mm) was generated to determine the pelvic orientation. Subsequently, a 200-mm unilateral dataset (layer thickness: 1 mm) was produced to analyze the anatomy of the hip and positioning of the implants. The knee and ankle were reconstructed using a field of view from layer thickness of 300 mm to 250 mm, respectively. Moreover, a layer thickness of 2 mm was used to determine the rotation of the femur and the length of the leg. The positioning of the implant was determined based on this 3D model of the lower limb.

**Fig. 1:**
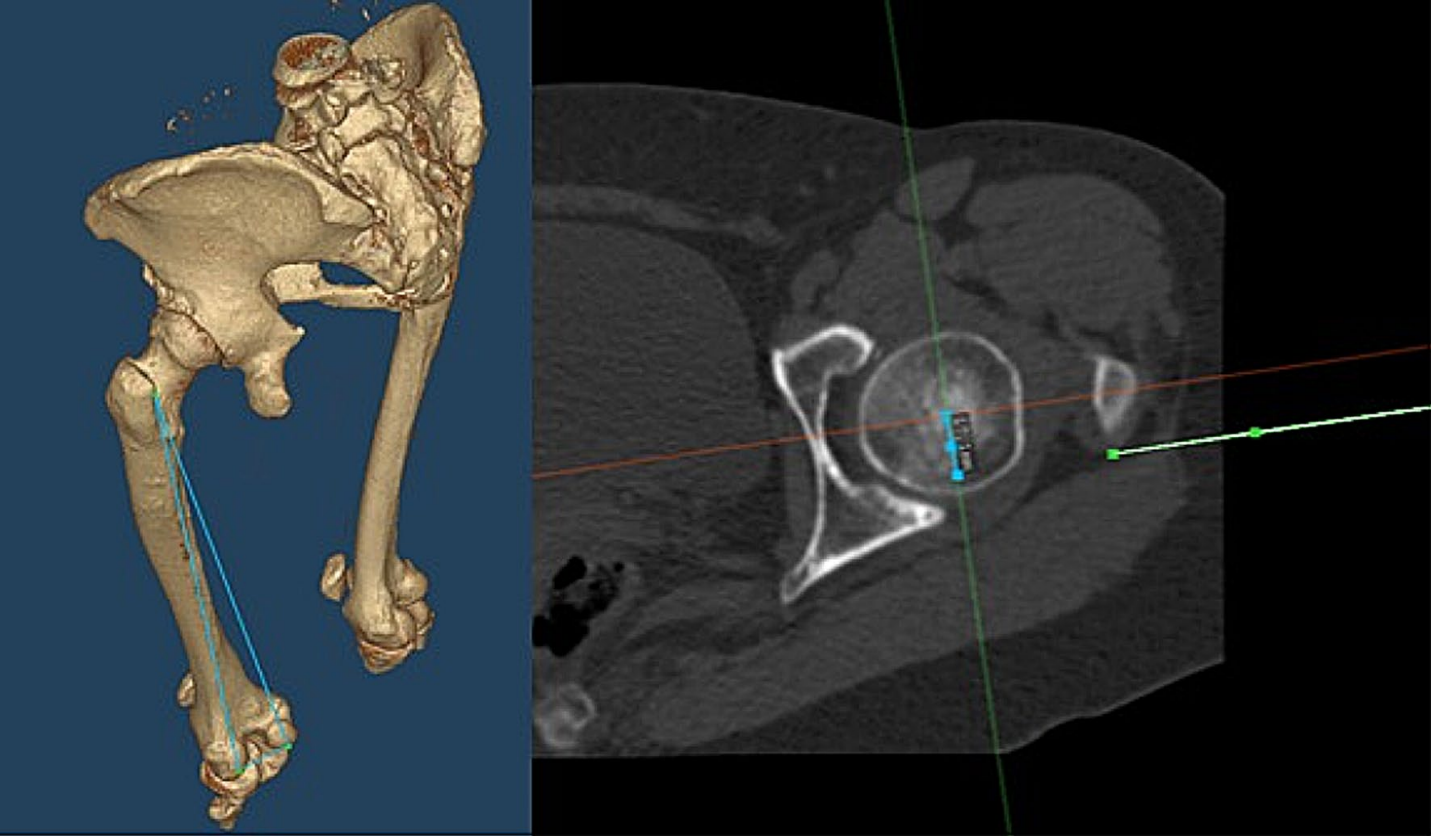
Definition of the AFO: perpendicular distance from the center of the femoral head (blue line on the right side) to a plane defined by the most posterior point of the medial and lateral femur condylus and the most posterior point of the trochanter major (blue triangle on the left side and green line on the right side)

The software generates a reference coordinate system based on the bony landmarks of the pelvis. This is necessary for the determination of several parameters, such as anatomic acetabulum/cup inclination and anteversion, COR, AFO, and LFO. Currently, there is no standard method available for the computation of the AFO. Hirata et al. previously proposed a method in which the distance from the center of the femoral head to the proximal femoral axis in the sagittal plane was measured [14]. However, this method depends on the anatomical antecurvation of the femur. We used a plane which contains three points of the femur: the most posterior point of the medial and lateral femur condylus and the most posterior point of the trochanter major. The AFO was defined as the perpendicular distance from the center of the femoral head to this plane (Fig. 1). In addition, the offset from the center of the acetabulum to the pelvis center was measured to determine the COR. Furthermore, the change in the craniocaudal direction was noted. The teardrop line served as a reference point.

Based on the parameters, the position and size of the implant was templated, aiming to reconstruct the anatomy. The templated sizes were implanted, and an anterolateral approach was used in all cases. The reaming procedure was based on Bonin’s anatomical technique. The cup was positioned below the subchondral bone [4]. Intraoperatively, a C-arch was used to position the cup. Postoperatively, a CT scan was performed for reevaluation, and the difference between the preoperative and postoperative values was determined (Fig. 3). The implants used in this study were the MobileLink^®^ cup and the cementless SP-CL^®^ stem (Waldemar LINK^®^, Hamburg, Germany). A descriptive analysis was performed owing to the limited number of cadaver specimens.

## Results

**Fig. 2:**
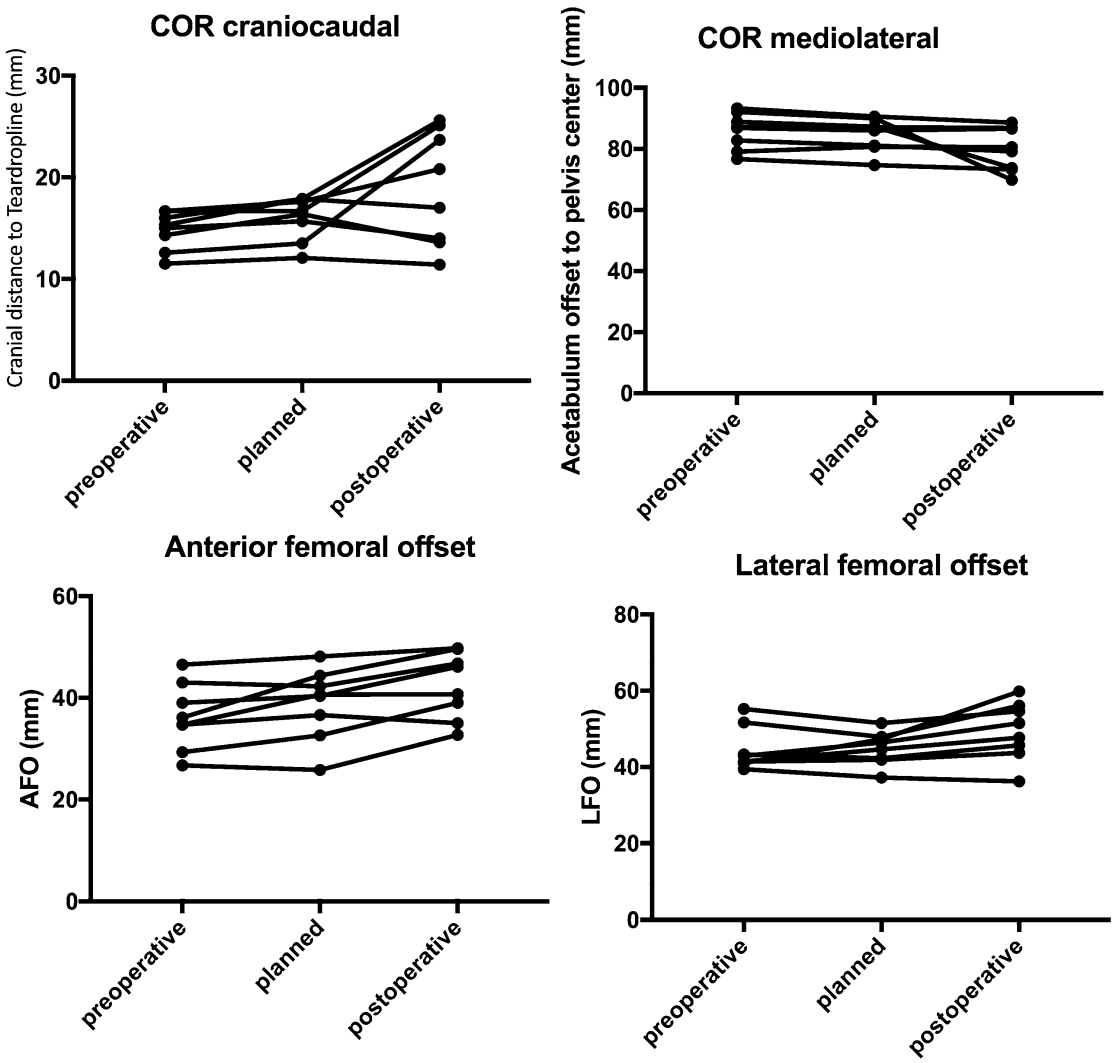
Visualization of the COR, AFO, and LFO for all specimens. In the COR graphs, the fractures of the acetabuli can be observed as the outliers, which were exceptionally increased and decreased postoperatively

Each implant was implanted with the predetermined size, without sizing problems. In one specimen, the biggest available size for the femur was insufficient. This fact was already noted during the preoperative planning. One specimen exhibited a preoperatively undetermined fracture in the right femoral neck and high-grade osteoporosis. In this case, both acetabuli were fractured during the surgery, due to bad bone quality. The medial wall was deficient with a central defect. Another acetabulum was fractured during implantation of the cup. However, there were no fractures observed in the contralateral side. The COR, LFO, as well as AFO were precisely reconstructed in all hips without fractures. The results for the anteversion and inclination are shown in Table 1. The results for the restoration of the COR in two planes and the determination of the lateral and AFO are shown in Table 2 and Fig. 2.

**Table 1:** Results for the anteversion and inclination of the cup/acetabulum in degrees

Cadaver	Anteversion				Inclination			
	Pre	Planned	Post	Delta	Pre	Planned	Post	Delta
No. 1 R	20.9	20.9	27.1	6.2	52.5	52.5	42	− 10.5
No. 1 L	21.7	21.7	43.6	21.9	51.7	51.7	54.1	2.4
No. 2 R	15.5	15.5	35.6	20.1	42.6	42.6	60.8	18.2
No. 2 L	13.7	13.7	47.4	33.7	42.3	42.3	36.8	− 5.5
No. 3 R	29.9	29.9	51.4	21.5	54.5	46	35.3	− 10.7
No. 3 L	29.3	25.6	31	5.4	47.9	44.6	53.2	8.6
No. 4 R	15.4	17.3	26.2	8.9	51.8	44.4	41.6	− 2.8
No. 4 L	14.1	15.4	18.2	2.8	51.2	45.8	45.3	− 0.5
Mean	20.06	20.00	35.06	15.06	49.31	46.24	46.14	− 0.10

**Table 2:** Results for the COR in the mediolateral and craniocaudal planes for the LFO and AFO in mm

Cadaver	COR mediolateral				COR craniocaudal				LFO				AFO			
	Pre	Planned	Post	Delta	Pre	Planned	Post	Delta	Pre	Planned	Post	Delta	Pre	Planned	Post	Delta
No. 1 R	88.9	87.3	73.8	− 13.5	12.6	13.5	23.7	− 10.2	41	47.3	59.8	12.5	26.7	25.8	32.7	6.9
No. 1 L	86.9	86	86.6	0.6	11.5	12.1	11.4	− 0.7	42.9	46.4	51.5	5.1	34.7	40.6	40.7	0.1
No. 2 R	79.1	80.7	80.6	− 0.1	14.3	16.4	13.6	− 2.8	41.4	44.6	47.7	3.1	39	40.4	46.1	5.7
No. 2 L	82.8	81.1	79.2	− 1.9	15	15.7	14	− 1.7	43.3	42.3	45.7	3.4	43	42.3	46.8	4.5
No. 3 R	92.1	90	69.9	− 20.1	16	17.9	25.6	− 7.7	39.4	37.2	36.2	− 1	46.5	48.1	49.8	1.7
No. 3 L	76.7	74.7	73.3	− 1.4	16.7	16.7	25.1	− 8.4	41.4	41.9	43.7	1.8	36.1	44.4	49.6	5.2
No. 4 R	87.1	87.1	86.9	− 0.2	16.7	17.6	20.8	3.2	55.2	51.5	54.6	3.1	34.7	36.6	35	− 1.6
No. 4 L	93.3	90.6	88.6	− 2	15.3	17.9	17	− 0.9	51.7	47.9	56.1	8.2	29.3	32.6	39	6.4
Mean	85.86	84.69	79.86	− 4.83	14.76	15.98	18.90	− 3.65	44.54	44.89	49.41	4.53	36.25	38.85	42.46	3.61

## Discussion

The most important finding of this study is the high accuracy of the preoperative templating using the 3D software. The planned implant size was always correct, as well as for the acetabulum and for the femur. Restoration of the anatomical COR was precisely achieved within 3 mm. The AFO was correctly restored, with a mean of 3.6 mm. The LFO exhibited a slightly higher mean of 4.53 mm.

**Fig. 3:**
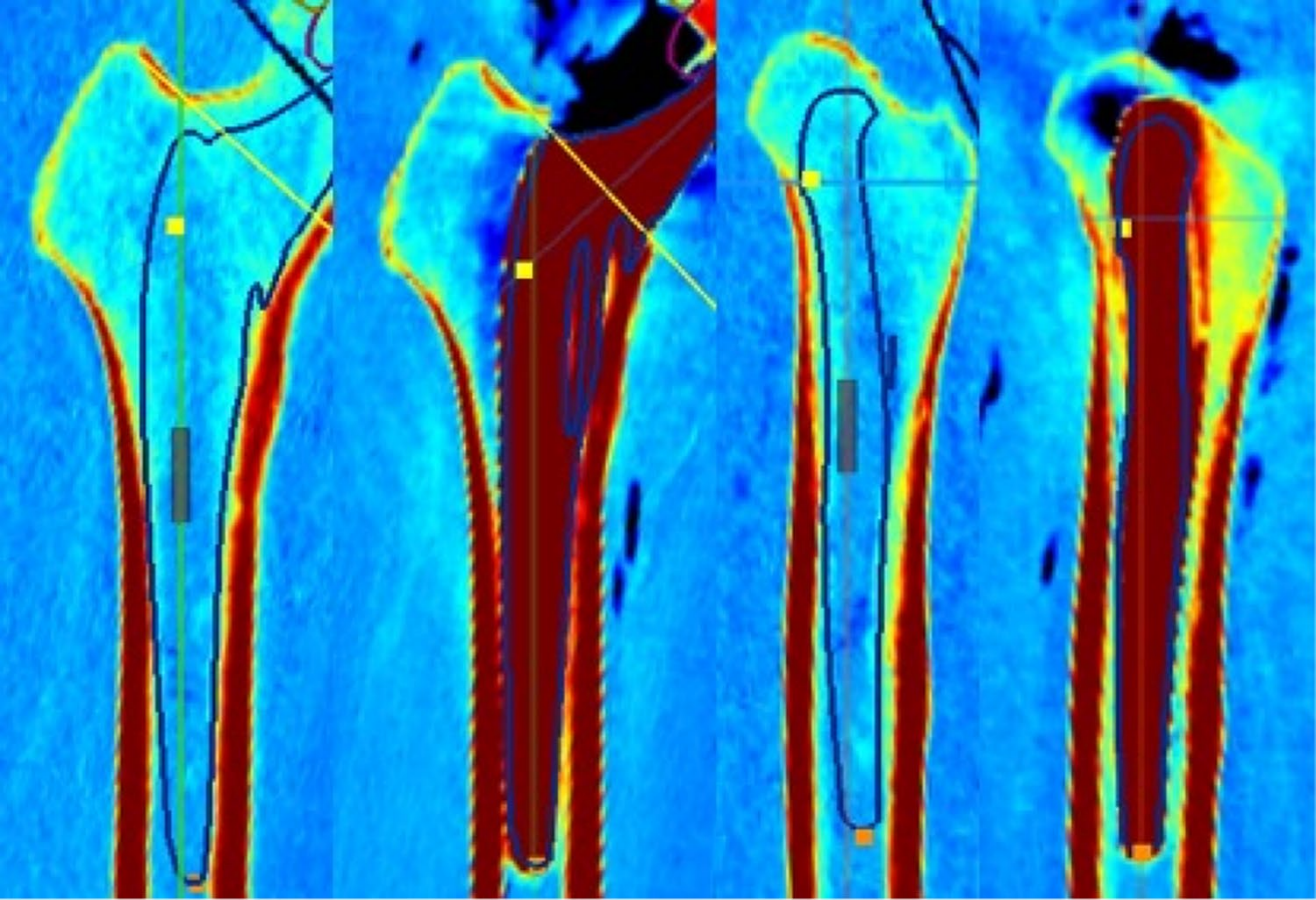
Templating of the stem in two planes with respect to the anatomical situation and the postoperative control of the results

The templated implant size of the femoral component was correct in all cases. Moreover, in cases in which the anatomy did not fit perfectly to the shape of the implant—as determined during the preoperative planning—the implantation was challenging. Notably, the software shows the exact anatomy (Fig. 3). Hence, inclusion of a safe zone between the implant and the corticalis—as performed in 2D templating—is not necessary [15]. The 3D templating helps to identify architectural hip deformities in order to adept the implant position to avoid soft tissue complications or instability [18]. In one specimen, the largest implant size of the femur was insufficient. In such cases, a different implant type should be selected. The largest SP-CL^®^ stem was size 16. Owing to the limited number of specimens, we still performed the procedure, despite the too small stem. Overall, the accuracy of the preoperative size planning was great, with a small learning curve. These findings are consistent with those previously reported in the literature [13, 30, 31]. According to the results, the bony anatomy of the femur specifies the exact size and position of the implant. In this case, navigation is only necessary for the correct positioning of the saw cut. Collectively, this evidence indicated that the development of TDI through the use of 3D templating is conceivable.

The role of the LFO in patient outcome is clear. The exact restoration of the LFO is associated with great benefits [3, 21, 22, 29]. Reduced polyethylene wear and dislocation rate, as well as improved clinical benefits (i.e., pain and abductor lever arm strength), are important points [3, 23]. However, the measurement technique using conventional X-ray is not accurate. Use of a CT scan with a 3D model offers advantages in terms of precision versus the conventional method. In our study, the mean preoperative LFO was 44.54 mm. Postoperatively, the mean LFO increased to 49.41 mm. A decrease in the LFO (i.e., 1 mm) was observed in only one case (s. Fig. 2 and Table 2). Bjordal and Bjorgul recommended to enlarge the LFO rather than reduce it [3]. In addition, Little et al. showed that an increase in the LFO by > 5 mm may lead to higher polyethylene wear [23]. Moreover, Sariali et al. confirmed that a decrease in the LFO by > 15% may lead to an alteration of the gait [29]. Overall, our data are in the recommended safe zone. However, the clinical influence of the AFO has not been sufficiently documented. The definition of the AFO is based on the findings reported by Mueller et al. Notably, the AFO depends on the stem anteversion and tilt [27]. Hirata et al. showed that the AFO correlated positively with the ROM of the hip [14]. In a 3D simulation study, they demonstrated an increase in higher flexion and internal rotation, with a simultaneous decrease in extension and external rotation. To the best of our knowledge, biomechanical or clinical data regarding this topic are sparse. Furthermore, the evidence regarding the measurement of this parameter is currently unclear.

Different methods, with advantages and disadvantages, have been developed. Hirata et al. defined the AFO “as the anterior distance from the center of the femoral head to the proximal femoral axis in the sagittal plane” [14]. The advantage of this method is that the values are comparable to those obtained from other patients or cohorts. However, the major disadvantage of this procedure is the limited accuracy in the measurement of the proximal femoral axis. This measurement is highly dependent on the anatomical ante-curvation of the femur. However, the most important factor in THA is the restoration of the individual anatomy. Therefore, the development of a method for the measurement and comparison of the preoperative and postoperative AFO is warranted. For this purpose, we recommend using the present measurement technique which is independent from the anatomical ante-curvation of the femur (s. in methods and Fig. 1). In this study, we reproduced the AFO with a mean failure of 3.6 mm (range − 1.6 to 6.9 mm). To the best of our knowledge, these values are the first of this kind to be reported. Hirata et al. postulated an optimal AFO for an optimal ROM of 15–25 mm. However, we cannot compare the present values with those previously reported. In the present study, the restoration of the AFO using the “modiCAS|3D^®^” software in cadavers was satisfactory (s. Fig. 2 and Table 2).

The COR is an important parameter in THA. Reconstruction of the COR during surgery offers several advantages. The main parameters for the COR are in the mediolateral and craniocaudal planes. Bonin et al. reported that a medialization > 5 mm occurs in 44% of cases treated with a conventional surgery technique (i.e., without consideration of the anatomy). Using an anatomical peripheral reaming technique, in which the cup was positioned at the level of the subchondral bone, the medial shift of the COR was 1.6 mm [4]. This improves the ROM and decreases bony impingement. Furthermore, a revision of the cup is easier, owing to the better bone stock [5, 31]. Using a conventional reaming technique, Meermans et al. showed similar results, with a medial and sagittal deviation of 5 mm and 3.7 mm, respectively. The anatomical technique provides an accuracy < 1 mm compared with the anatomy [26]. In other studies investigating the manual technique, an accuracy of approximately 5 mm was reported [2]. Surprisingly, the acetabular offset was reproducible with navigation in THA within 6 mm and 8 mm, respectively, in 95–98% of cases [10, 35]. Dastane et al. suggested that the COR should be restored < 3 mm mediolateral and 5 mm craniocaudal to avoid the negative aspects [9]. In this study investigating an anatomical freehand reaming technique, the mediolateral and craniocaudal deviations were < 2 mm and < 3.5 mm, respectively, in the absence of fracture of the acetabulum (see Fig. 2 and Table 2). It is important to emphasize that we did not have any intraoperative control options for the positioning of the cup, except for a C-arch. In most cases, the COR was slightly medialized and caudalized. In addition, the obtained values were in the recommended safe zone. During surgery, the optimal inclination and anteversion of the cup has no top priority in this study since the adjustment of the cup concerning these parameters was quite difficult because of the limited mounting of the specimens. Therefore, these findings should be interpreted cautiously.

The main limitation of this study was the lack of a control mechanism for the positioning of the implant (e.g., navigation or a robotic system), especially for the preparation of the cup. The milling does not have to be oriented to a boundary of the cortical bone, as in the case of femoral preparation. Therefore, the exact positioning is not guaranteed. This should be the focus of subsequent studies. Another limitation of the present study was the small sample size. One cadaver exhibited major osteoporosis, which led to fracture of both acetabuli. Therefore, the results for these cups could not be evaluated. Furthermore, one other fracture occurred due to a brittle half-frozen bone stock. Subsequently, an operation was performed in the contralateral side, without problems in the implantation of the cup. Overall, fracture was in our opinion not a problem due to excessively large implant.

The results obtained for the AFO cannot be compared with those previously reported due to the different measurement methods used in the studies. In addition, determination of the length of the leg was not possible. The CT scan was performed in frozen cadavers, with a not standardized leg position (e.g., flexion in the knee joint). The evaluation of this parameter in future studies may also yield interesting results.

## Conclusion

Anatomic reconstruction is possible using CT-based templating software. TDI is possible according to the findings of this study. However, additional technical aids (e.g., navigation or robot-assisted surgery) are required to implement the preoperative plan and increase the accuracy.
